# Microstructure Evolution in a 6082 Aluminium Alloy during Thermomechanical Treatment

**DOI:** 10.3390/ma11081319

**Published:** 2018-07-30

**Authors:** Cecilia Poletti, Romain Bureau, Peter Loidolt, Peter Simon, Stefan Mitsche, Mirjam Spuller

**Affiliations:** 1 Institute for Materials Science, Joining and Forming, Graz University of Technology, Kopernikusgasse 24, 8010 Graz, Austria; 2 Advanced Materials and Mechanical Testing, French-German Research Institute of Saint-Louis, 5 rue du Général Cassagnou, 68300 Saint-Louis, France; romain.bureau@isl.eumailto; 3 Institute for Process and Particle Engineering, Graz University of Technology, Inffelddgasse 13/3, 8010 Graz, Austria; peter.loidolt@tugraz.at; 4 AMAG Austria Metall AG, Lamprechtshausenerstrasse 61, P.O. Box 3, 5282 Braunau-Ranshofen, Austria; peter.simon@amag.at; 5 Institute of Electron Microscopy and Nanoanalysis of the TU Graz (FELMI), Graz Centre for Electron Microscopy (ZFE Graz), Steyrergasse 17, 8010 Graz, Austria; smitsche@tugraz.at; 6 Erich Schmid Institute of Materials Science of the Austrian Academy of Sciences, Jahnstrasse 12, 8700 Leoben, Austria; mirjam.spuller@oeaw.ac.at

**Keywords:** thermomechanical treatment, aluminium alloy, recovery, recrystallisation, dislocations, materials modelling

## Abstract

Thermomechanical treatments of age-hardenable wrought aluminium alloys provoke microstructural changes that involve the movement, arrangement, and annihilation of dislocations, the movement of boundaries, and the formation or dissolution of phases. Cold and hot compression tests are carried out using a Gleeble^®^ 3800 machine to produce flow data as well as deformed samples for metallography. Electron backscattered diffraction and light optical microscopy were used to characterise the microstructure after plastic deformation and heat treatments. Models based on dislocation densities are developed to describe strain hardening, dynamic recovery, and static recrystallisation. The models can describe both the flow and the microstructure evolutions at deformations from room temperatures to 450 °C. The static recrystallisation and static recovery phenomena are modelled as a continuation of the deformation model. The recrystallisation model accounts also for the effect of the intermetallic particles in the movements of boundaries.

## 1. Introduction

The production process of 6xxx series aluminium sheets consists in a succession of thermomechanical steps designed to improve the strength of the product while reaching the desired geometry. The initial billet with its specific chemical composition is produced by continuous or batch casting. Each subsequent step brings irreversible changes in the microstructure that directly affect the mechanical properties of the material. A combination of recrystallised, finely grained, and precipitation hardened microstructure brings the best mechanical strength to the sheet while preserving a reasonable ductility for further shaping processes.

It is now a well-established practice to model the industrial processes with finite element methods, which require material data as an input. Modelling allows to roughly calculate the properties of the final product, supporting the design and optimisation of the production processes. As the models can only be as good as our understanding of the physical phenomena they are meant to represent, an experimental investigation is always needed to validate their output and to understand the underlying physical phenomena.

Phenomenological models consist in setting up a constitutive equation linking the flow stress to the strain, the strain rate, and the temperature, and optimising it so that it best represents the main features of the flow curves. The equation usually features a power law dependency for the strain and the strain rate, and an activation energy for the thermal dependency [1,2,3,4]. Such models present the advantages of being easy to set up and requiring almost no computational power, but they do not provide any insight on the physics of the problem at hand.

Physical models are more deeply connected to the microstructure evolution. The modification of the microstructure depends essentially on the material itself, its initial state, and its thermomechanical history. In the last decades, metallurgists have developed models to describe the strain hardening of metallic alloys during forming processes using physical-based microstructural approaches. Such approaches usually consist of three main features [5,6,7,8]; a set of independent internal variables representative of the microstructure (classically dislocation densities, subgrain sizes, precipitation state, etc.), the evolution rates of these variables, and a constitutive equation to link the microscopic variables with the flow stress of the material. The difficulty in observing the underlying mechanisms responsible for the variable evolution often leads to the appearance of a large amount of model parameters [7,8].

During deformation, the microstructure of metallic materials develops permanently, leading to important dynamic variations in the macroscopic stress required to further deform the material. Strain hardening, for example, results directly from the multiplication of microscopic defects such as dislocations. These variations essentially depend on the strain, the strain rate, and the temperature of work. After deformation, for example in between passes in a rolling process, the microstructure may undergo static recovery, that is, the annihilation and rearrangement of microstructural defects that also affects the flow stress of the material. Flow stresses developed during hot rolling at elevated temperatures can be modelled using the total dislocation density as a single internal variable to represent the microstructure [6], but modelling the behaviour of aluminium alloys from room to moderate temperatures requires at least two kinds of internal variables [6]. In rolled aluminium products, static recrystallisation occurs after cold rolling during a recrystallisation or a solution treatment. The dominant mechanism for the nucleation new grains is strain-induced boundary migration [9,10], whereby subgrains lying on the boundaries of existing deformed grains bulge into the neighbouring grain and grow further after reaching the critical size for nucleation. As the motion of boundaries is a diffusional process, it is influenced by the temperature. The strain grade directly influences the number of potential nuclei being available for further growth.

In this work we propose a physically based constitutive model applicable to cold and hot working over a wide range of strain rates using three internal variables. Two kinds of dislocation densities that can evolve are responsible for the strain hardening, while the third kind accounts for the deformation and can be derived directly from the Orowan equation [11]. Simple evolution rates are split in a dynamic part and a static part, enabling static recovery. A subsequent recrystallisation model featuring the same internal variables was developed that considers the level of strain reached during earlier deformation and encompasses the competition between recrystallisation and static recovery. The physical phenomena were derived and validated from experimental results obtained during cold and hot deformation and further recrystallisation of a 6082 aluminium alloy.

## 2. Materials and Methods

### 2.1. Material

A commercial 6082 aluminium alloy with the chemical composition shown in Table 1 was studied. The material was delivered after hot rolling into a plate of thickness 3.9 mm and subsequent air cooling at room temperature. No homogenisation treatment was applied before rolling.

### 2.2. Experimental Methods

Samples of size 10 mm in the rolling direction and 20 mm in the transverse direction were cut out of the plate and compressed down to a thickness of 1.5 mm in plane strain condition using a Gleeble^®^ 3800 machine (Dynamic Systems Inc., 323 NY 355 Poestenkill, NY, USA). The experiments were carried out between 25 °C and 400 °C, at strain rates of 0.01, 0.1, 1, and 10 s^−1^. The temperature was controlled by a J type thermocouple welded on the surface of the samples. Samples deformed at room temperature and 10 s^−1^ were then annealed in an oven at 300 °C and 400 °C for 10 s, 1 min, 5 min, and 1 h to induce recrystallisation.

Light optical microscopy was carried out to determine the grain shape and size. Polarised light was used after metallographic preparation of the sample with Barker’s reagent (5 mL HBF4 48%vol in 200 mL water). Additionally, the precipitates were characterised in scanning electron microscope (SEM) Zeiss Ultra 55 (Carl-Zeiss AG, Oberkochen, Germany) with the use of a backscattered electron detector (BSE) which shows material contrast, and an energy dispersive X-ray detector (EDS) EDAX Genesis (EDAX Business Unit AMETEK GmbH, Weiterstadt, Germany). For these characterisations, the SEM was operated at a primary beam energy of only 6 kV to gain high surface sensitive measurements. The intermetallic phases were detected using BSE detector. The density $N_{S}$ of particles intersecting the sample surface, as well as the average area of intersection $\bar{A_{P}}$ of individual particles, were readily measured from the obtained micrographs. Assuming many randomly distributed spheres of radius $R_{P}$, the averaging of the area of intersection of individual particles with the sample surface over $\left[-R_{P};R_{P}\right]$, is written as:

(1) $$\bar{A_{P}}=\frac{2\pi }{3}R_{P}^{2}$$

For a homogeneous distribution of particles, the volume fraction of particles $F_{V}$ reads:

(2) $$F_{V}=\frac{2\pi }{3}R_{P}^{2}N_{S}=\bar{A_{P}}N_{S}$$

The as-received, deformed, and annealed samples were investigated by electron backscattered diffraction (EBSD) coupled with EDS. These investigations provided information about the grain and subgrain structure and of the intermetallic phases. All coupled EBSD–EDS investigations were performed at a primary beam energy of 20 kV on the Zeiss Ultra equipped with an EBSD system from EDAX-TSL. A tolerance angle of 11° was used to determine the subgrain structure.

Finite element simulations of the plane strain tests were carried out with the software DEFORM^TM^ 2D (Scientific Forming Technologies Corporation, Columbus, OH, USA) to obtain the distribution of the equivalent strain values. The recrystallisation grade and the recrystallised grain size were characterised in the regions of strain 1 and 1.5 for both annealing temperatures. Grain orientation spread (GOS) maps were produced from EBSD measurements to distinguish recrystallised from non-recrystallised grains [12] defining a maximum spread limit of 3°.

### 2.3. Modelling Methods

#### 2.3.1. Microstructure Representation

The microstructure is assumed to be composed of well-defined subgrains, whose walls and interiors are populated with dislocations of respective densities $\rho_{w}$ and $\rho_{i}$. It is emphasised that, although $\rho_{w}$ usually stands for the dislocation density within the subgrains [7,8], it represents here the total length of wall dislocation per unit volume of the material. The later definition yields lower densities than the former. Additionally, mobile dislocations of density $\rho_{m}$ travel across several subgrains before being stored in some manner, accounting for the macroscopic strain. The total density of dislocations $\rho_{t}$ hence reads:

(3) $$\rho_{t}=\rho_{w}+\rho_{i}+\rho_{m}$$

The subgrain size δ in the deformed material can be calculated out of the dislocation densities. Two methods are available in the literature [9]. If the subgrain boundaries are assumed to be tilt boundaries and if the wall dislocation density is averaged over the microstructure, then:

(4) $$\delta =\frac{\kappa \bar{\theta }}{b\rho_{w}}$$

κ is a shape factor and $\bar{\theta }$ is the average crystal orientation difference between each side of the boundary. This approach works fine at low to intermediate temperatures. 

#### 2.3.2. Constitutive Equation

The governing constitutive equation is chosen to have the following form:

(5) $$\;\sigma =M\left(\tau_{ath}+\tau_{eff}+\tau_{d}\right)\;$$

where σ is the flow stress of the material and M is the Taylor factor, accounting for the polycrystalline nature of the material [13]. The athermal shear stress $\tau_{ath}$ resolved on the slip plane translates the long-range interaction of dislocations via their elastic strain field, and reads [14]:

(6) $$\;\tau_{ath}=\alpha \mu b\sqrt{\rho_{t}}\;$$

α is a stress constant, μ is the temperature dependent shear modulus, and *b* is the Burgers vector. The effective resolved shear stress $\tau_{eff}$ is the additional stress required for mobile dislocations to be able to cut through the forest of dislocations cutting the slip plane and hindering them locally on their way through the microstructure. It is given by:

(7) $$\tau_{eff}=\frac{Q}{V}+\frac{k_{B}T}{V}\exp\left(\frac{v_{m}}{L\nu_{D}}\right).\;$$

L is the mean free path of dislocations, $\nu_{D}$ is the Debye vibrational frequency of the material, Q is the energy barrier of forest dislocations, V has the dimension of a volume and is classically referred to as the activation volume, $k_{B}$ is the Boltzmann constant, and *T* is the temperature. The glide velocity $v_{m}$ of mobile dislocations is given by the Orowan equation:

(8) $$M\dot{\epsilon }=\rho_{m}bv_{m}$$

where $\dot{\epsilon }$ is the rate of strain. The contribution of the intermetallic phases to the resolved shear stress $\tau_{d}$ is the Orowan stress [15]:

(9) $$\tau_{d}=\frac{\mu b\sqrt{V_{d}}}{R_{d}}$$

$V_{d}$ and $R_{d}$, respectively, being the volume fraction of dispersoids and their equivalent radius.

#### 2.3.3. Rate Equations

The evolution rates of $\rho_{i}$ and $\rho_{w}$ are each given by a Kocks–Mecking type of equation [16] supplemented by a static annihilation term [17]:

(10) $$\;\frac{\partial \rho_{x}}{\partial t}=\dot{\epsilon }\left(\frac{h_{1,x}}{b}\sqrt{\rho_{x}}-h_{2,\;x}\rho_{x}\right)-h_{3,x}D{\left(\rho_{x}-\rho_{x,eq}\right)}^{2}$$

with x=i,w. $h_{1,x}$, $h_{2,x}$ and $h_{3,x}$ are dimensionless model parameters. $\rho_{x,eq}$ is an equilibrium dislocation density of a fully recrystallised material. D is the diffusion coefficient:

(11) $$\;D=b^{2}\nu_{D}\exp\left(-\frac{Q_{self}}{k_{B}T}\right)\;$$

where $Q_{self}$ is the activation energy for self-diffusion. In Equation (10), the evolution rates are split into a dynamic part, linked to the strain rate, and a static part, diffusion driven. Although the kinetics of static mechanisms are negligible with respect to dynamic mechanisms, such a form of the model allows for diffusional phenomena when the strain rate is low or null and the temperature high enough.

#### 2.3.4. Recrystallisation Model

The driving force for static recrystallisation and static recovery being related to the local stored energy, both processes happen simultaneously and competitively during annealing. The dislocation density decrease due to static recovery can be calculated by setting $\dot{\epsilon }=0$ in Equation (10). If the subgrain growth is assumed to be driven by capillarity [9]:

(12) $$\;\frac{\partial \delta }{\partial t}=M_{s}\frac{1.5\gamma_{s}}{\delta }.\;$$

$M_{s}$ being the mobility of low angle grain boundaries and $\gamma_{s}$ their specific energy, given by a Read and Shockley relationship [18] of the form:

(13) $$\;\gamma_{s}=\frac{\mu b\theta }{4\pi \left(1-\nu \right)}\ln\left(\frac{e\theta_{c}}{\theta }\right)\;$$

where $\theta_{c}$ is the critical orientation difference for a low angle boundary to turn into a high angle boundary, e is the natural exponential, and ν is the Poisson coefficient of the material. Since the mobile dislocation density is negligible with respect to the subgrain interior dislocation density, the difference in stored volume energy ΔE between non-recrystallised and recrystallised grains reads:

(14) $$\;\Delta E=0.5\mu b^{2}\rho_{i}+\frac{1.5\gamma_{s}}{\delta }\;$$

Capillarity also tends to promote the growth of recrystallised grains, which translates in a driving pressure $P_{C}$:

(15) $$\;P_{C}=\frac{1.5\gamma_{g}}{D}\;$$

where $\gamma_{g}$ is the specific energy of high angle grain boundaries and D is the mean grain size. Second phase particles hinder the movement of boundaries by exerting a retarding pressure $P_{Z}$ on them. The Zener mechanism [19] yields the following equation:

(16) $$\;P_{Z}=\frac{3\gamma_{g}V_{d}}{2R_{d}}\;$$

The total driving pressure for recrystallisation P is then classically given by:

(17) $$\;P=\Delta E+P_{C}-P_{Z}\;$$

The growth rate of recrystallised grains $\dot{G}$ can be written as:

(18) $$\;\dot{G}=M_{g}P\;$$

$M_{g}$ being the mobility of high angle grain boundaries. The grain radius is then calculated by integrating Equation (18) over time.

(19) $$\;D=2\int_{t_{0}}^{t}\dot{G}\left(t^{\prime }\right)dt^{\prime }\;$$

The factor 2 accounts for the fact that D is the diameter of recrystallised grains. The incubation time $t_{0}$, that is, the time it takes for the nuclei to reach the critical size and start growing, is temperature dependent and must be adjusted. According to the JMAK theory [20], the extended recrystallised volume can be calculated as the product of the number N of nuclei available in the microstructure with the volume of a recrystallised grain. For site saturated nucleation, meaning that the N nuclei are created during deformation prior to annealing, this yields an equation of the type:

(20) $$\;V_{ext}=NfD^{n}\;$$

where f is a shape factor and n the Avrami exponent. The fraction of recrystallised material X is then given by:

(21) $$\;X=1-\exp\left(-V_{ext}\right)\;$$

The number of nuclei reaching the critical size for nucleation is written as follows:

(22) $$\;N=N_{0}\epsilon \exp\left(-\frac{\gamma_{g}b^{2}}{k_{B}T}\right)\;$$

where $N_{0}$ is a model parameter. The activation energy is taken as the product of the specific energy of high angle boundaries with a typical area, as for strain induced boundary migration to happen, subgrains have to bulge into the neighbouring grain and displace the existing boundary. Grain growth after recrystallisation is driven only by capillarity. Hence, the driving pressure is the same as in Equation (17), without the term ΔE. The grain size is again given by integrating the growth rate, but now starting at the time of end of recrystallisation.

#### 2.3.5. Parameter Initialisation

The parameters of the flow stress model were initialised as follows: α=0.5, b=0.286 nm [21], M=3.06 [22], $\nu_{D}=1.5\times 10^{13}$ s^−1^, ν=0.33, and $Q_{self}=0.98$ eV. The shear modulus reads $\mu =\left(84.8-4.06\times 10^{-2}T\right)/\left(2\left(1+\nu \right)\right)\;$ in GPa (temperature in K) [23]. The microstructure being initially fully recrystallised, $\rho_{i}$ and $\rho_{w}$ were initially taken equally low and equal to $10^{10}$ m/m^3^. Since the model must be able to work out the yield stress of the material, a least square optimisation method was run on $\rho_{m}$, $V_{act},$ and Q to best capture the temperature and strain rate dependency of the yield stress, with $\rho_{m}$ let free to vary with the strain rate. The following values were worked out: $\frac{\rho_{m}}{\rho_{m}^{*}}=e^{2.28}{\left(\frac{\dot{\epsilon }}{{\dot{\epsilon }}^{*}}\right)}^{0.65}\;$1/m^2^, $V_{act}=1-50\times 10^{-27}$ m^3^, and Q=1.44 eV. The rate parameters $h_{1,x}$ and $h_{2,x}$ (x=w, i) were optimised to best capture the strain hardening of the material, while the $h_{3,x}$ (*x* = *i*, *w*) were taken equal to 1.

The parameters of the recrystallisation model were initialised as follows: $\gamma_{g}=0.324$ J·m^−2^ [9]. It was assumed that the subgrain misorientation already reaches, at very small strain values, a mean value of θ=3° [8]. The critical misorientation was selected as $\theta_{c}$ = 15°. $M_{g}$ classically has an Arrhenius expression of the form $M_{g}=M_{0}\exp\left(-Q_{g}/k\_BT\right)\;$ and the literature provides wide ranges of values for both $M_{0}$ and $Q_{g}$. The following was determined from our recrystallisation experiments and used here: $M_{0}=3.1\times 10^{-9}$ m^4^·J^−1^·s^−1^ and $Q_{g}=0.50$ eV. The mobility of the low angle grain boundaries $M_{s}$ was taken equal to $0.02\;M_{g}$ [24]. The parameter $N_{0}$ was equal to $7\times 10^{10}$.

## 3. Results

### 3.1. Microstructural Features

#### 3.1.1. As-Received Material

**Figure 1:**
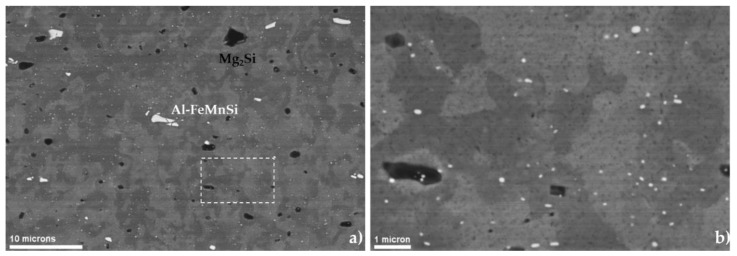
Microstructure of the as-received material observed by means of scanning electron microscopy (SEM) in backscattered electron mode (BSE) showing Al-FeMnSi (large and small) in white, and Mg_2_Si in black. (**a**) Overview and (**b**) detail of rectangle shown in (**a**).

Figure 1 shows the microstructure of the AA6082 material in the as-received condition, that is, hot rolled and air cooled. The microstructure consists of disc-like grains partially recrystallised.

Three types of intermetallic phases were identified: 1 vol % of stable *β*-phase (Mg_2_Si) with a mean radius of 0.35 µm, a population of large Al-FeMnSi phases of 1.7 vol % with a mean radius of 2.5 µm, and 0.53 vol % of Al-FeMnSi phases with a mean radius of 60 nm. Only this last population of Al-FeMnSi was considered to produce strengthening by Orowan mechanism and therefore, $V_{d}$ = 0.53 vol % and $R_{d}$ = 60 nm were used in Equations (9) and (16). The other particles could not produce strengthening due to the low amount and large size. It is assumed that large Mg_2_Si existed only in the stable form due to the slow cooling after rolling.

#### 3.1.2. Plastically Deformed

**Figure 2:**
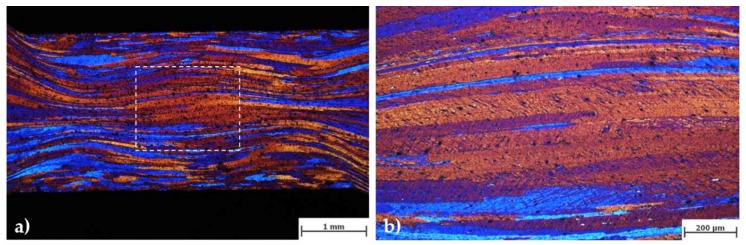
Elongated grains (**a**) and deformation bands (**b**) observed in the plane strain samples after cold deformation (optical microscopy). Nominal strain = 0.7.

Figure 2 shows the microstructure, the strain, and the hardness distribution within a plane strain sample after cold deformation. Elongated grains and substructure formation can already be detected with light optical microscopy. The deformation under non-frictionless conditions provoked the appearance of a double cross of strain concentration. 

**Figure 3:**
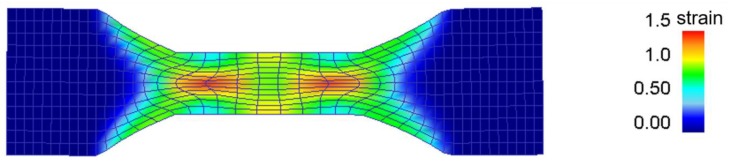
Finite element simulation using DEFORM^TM^ 2D showing the strain distribution within the plane strain sample after cold deformation at a nominal strain of 1.

The finite element calculations (Figure 3), show that the nominal strain of 1 is achieved in between the deformation crosses, whereas a local strain of 1.5 is achieved in the middle of each cross.

**Figure 4:**
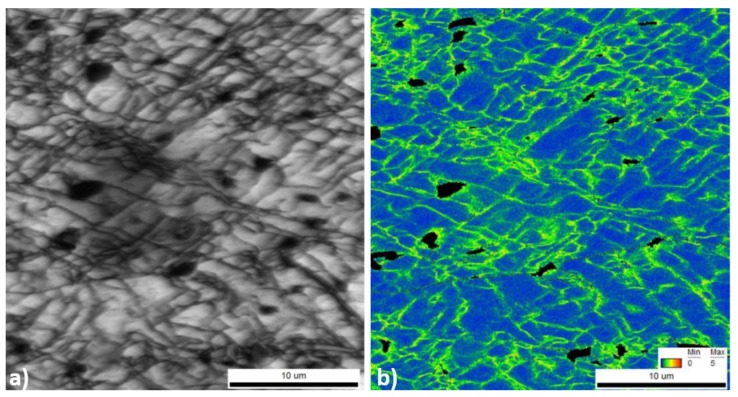
Microstructure of AA6082 after cold deformation determined by electron backscattered diffraction (EBSD) showing the cells in the (**a**) image quality (IQ) as well as (**b**) in the Kernel map. Black non-indexed areas represent the intermetallic positions.

The substructure developed after plane strain deformation at room temperature, as well as the location of the intermetallic phases, are shown in Figure 4. The formed cells have a mean diameter of 1.5 µm and are surrounded by high angle and low angle grain boundaries. Cell size is heterogeneous within a grain and among grains.

**Figure 5:**
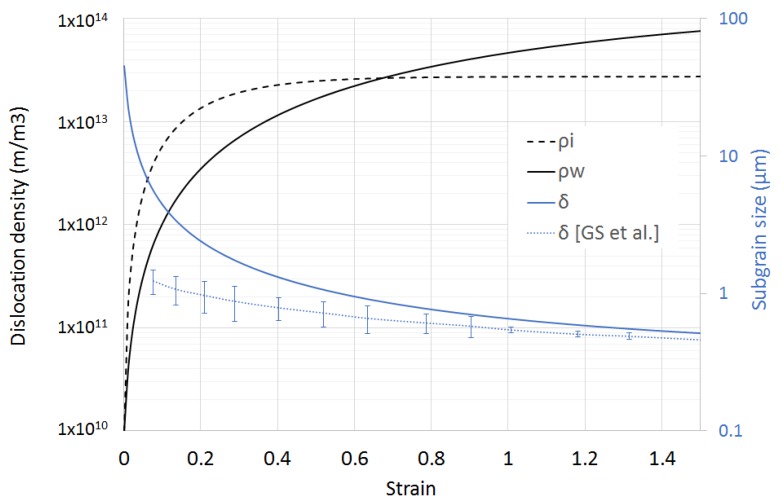
Modelled dislocation densities and subgrain size developed during deformation at room temperature and 10 s^−1^ of strain rate. Experimental data from Reference [25] show good correlation with the literature.

The evolution of the dislocation densities and the subgrain size at room temperature are shown in Figure 5. The steady hardening occurring during cold deformation is produced by the increment of the total dislocation density. While the dislocation densities in the subgrain interiors saturate at small strains, the wall dislocation density keeps increasing. The latter can be interpreted as a continuous decrease in the subgrain size, since the model assumes saturation of the misorientation between subgrains at 3°. The experimental data from Gil Sevillano et al. [25], as well as the own measured data point are in good agreement with the modelled data.

**Figure 6:**
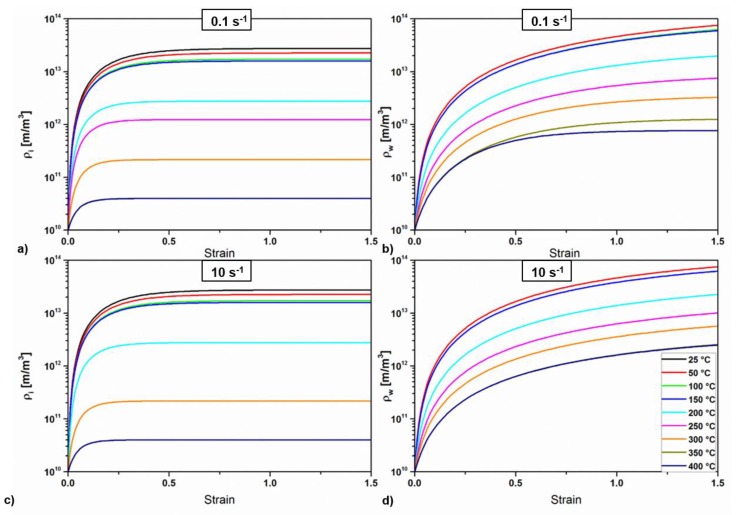
Evolution of the dislocation densities (modelled) as a function of temperature and for two selected strain rates. (**a**,**b**) interiors and walls dislocation densities, respectively, during deformation at 0.1 s^−1^, and (**c**,**d**) interiors and walls dislocation densities, respectively, during deformation at s^−1^.

The modelled cell interior and cell wall dislocation are shown in Figure 6. In general, it can be observed that $\rho_{i}$ saturates very rapidly, especially by increasing the temperature, although with very low influence of the strain rate in the levels of the calculated values and in the investigated range of strain rates. The wall dislocation density saturates at low strain rates and high temperatures due to a large dynamic recovery, while it keeps increasing when the strain hardening dominates. 

**Figure 7:**
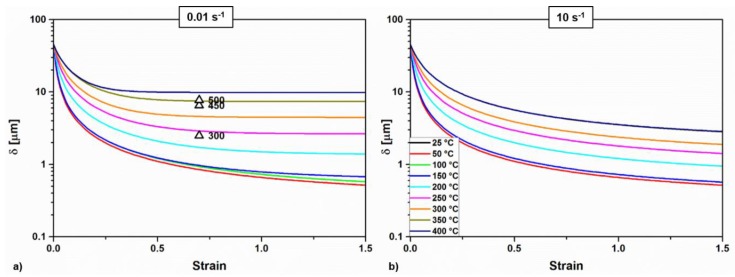
Calculated subgrain size evolution as a function of temperature and for two strain rates: 0.01 (**a**) and 10 s^−1^ (**b**). Comparison with experimental data from Reference [26].

In agreement with the evolution of the wall dislocation densities, the subgrain size (Figure 7) reaches a plateau at high temperatures and low strain rates, when the subgrains are predicted to be the largest. The results agree with subgrain size of a similar AA6082 material determined experimentally by EBSD after hot compression tests [26].

#### 3.1.3. Recrystallisation

**Figure 8:**
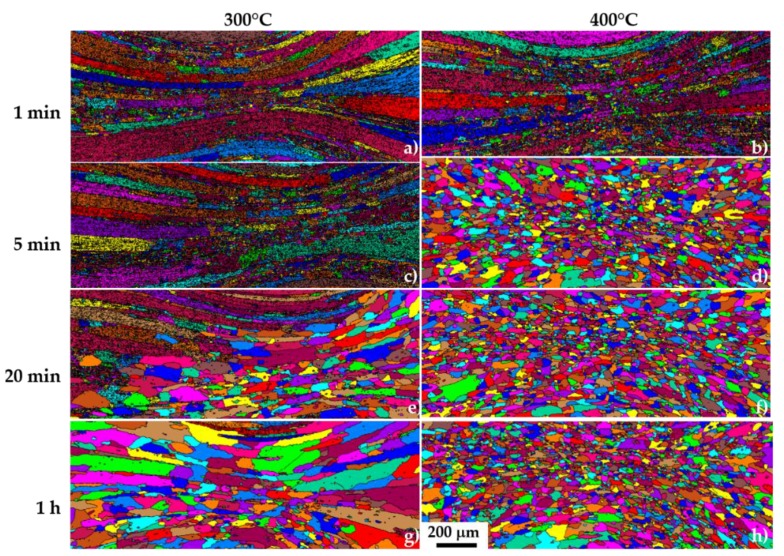
Unique grain colour map of the samples annealed at 300 °C (**a**,**c**,**e**,**g**), and 400 °C (**b**,**d**,**f**,**h**), for 1, 5, 20, and 60 min, respectively, obtained after EBSD measurements. The scale bar is applied to all sub-figures.

The recrystallisation behaviour of the material was studied after cold deformation in the regions of strain 1 and 1.5 for annealing at 300 °C and 400 °C. Unique grain colour maps obtained from EBSD data are shown in Figure 8. Recrystallisation was not observed in the samples treated at 300 °C before 20 min, and even then, only in the region of the highest strain. The microstructure is completely recrystallised after 1 h of annealing. The grains are smaller in the regions of larger strain, where the number of nuclei of recrystallised grains is higher. Elongated recrystallised grains were observed in the region of lower strain. The grain boundary migration is stopped by the Fe/Mn rich aluminides aligned in the direction of deformation. The microstructure is completely recrystallised after 5 min of annealing in the samples annealed at 400 °C, and no further grain growth is observed. The mean grain size after recrystallisation is larger at 300 °C than at 400 °C, which can be explained as follows: the critical size for nucleation is reached sooner at 400 °C than at 300 °C, leading to a larger number of nuclei. Their boundaries impinge rapidly upon each other, preventing further grain growth.

**Figure 9:**
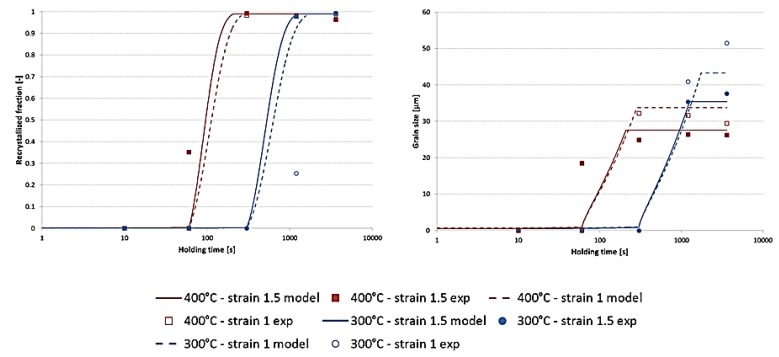
Recrystallised fraction (**left**) and grain size (**right**) during annealing following cold deformation at two different strains.

The evolution of the recrystallised fraction during annealing at 300 °C and 400 °C is given in Figure 9. The effect of the cold deformation is reproduced by the model: a larger strain results in a faster recrystallisation and smaller grains. A good agreement is found between the model and the experimental results. The grain size reaches a constant value due to a complete Zener pinning of the high angle grain boundaries by the larger Al-FeMnSi phases. The plateau is reached earlier when the annealing temperature is 400 °C.

### 3.2. Flow Stresses

**Figure 10:**
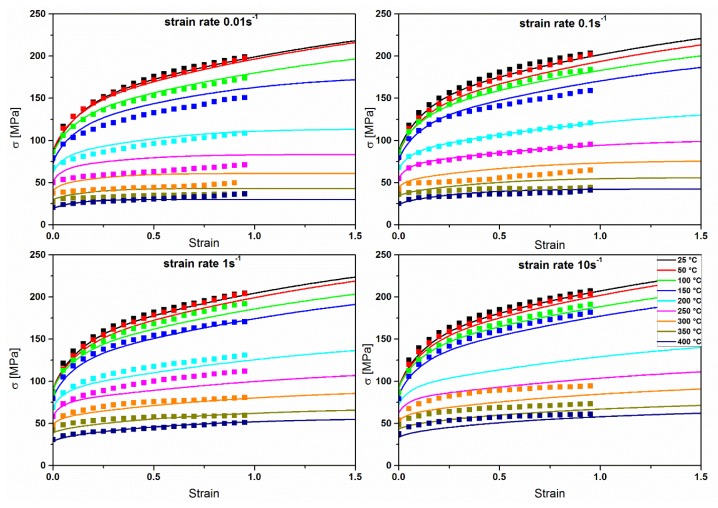
Measured (squares) and modelled (lines) flow stress at different strain rates and for temperatures ranging from 25 °C to 400 °C.

Experimental and modelled flow stresses are drawn in Figure 10. The modelled flow stresses show a good agreement with the experimental ones. A typical behaviour for aluminium alloys is observed: larger stresses at lower temperatures and higher strain rates, large strain hardening at low temperatures, and an increasing strain rate sensitivity by increasing the temperature. Although the model catches the temperature and strain rate effect on the stresses, in general the steady state is reached before than in the experimental data.

**Figure 11:**
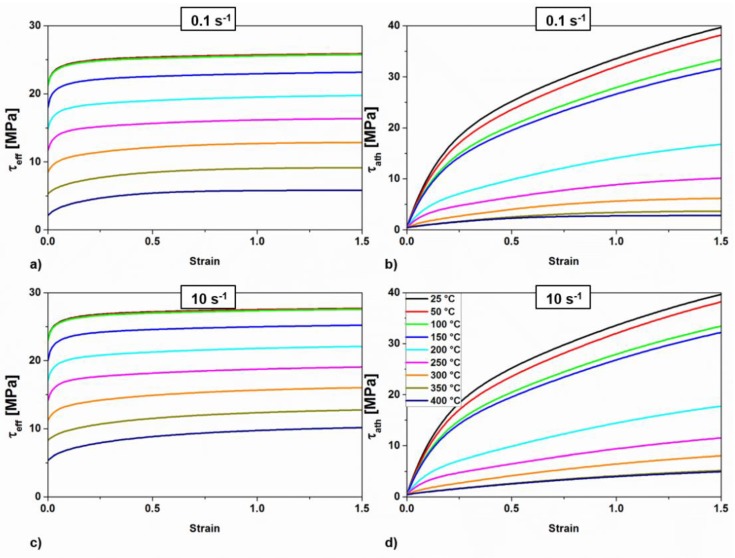
Effective and athermal stresses calculated for all temperatures and (**a**,**b**) 0.1 s^−1^ and (**c**,**d**) 10 s^−1^.

The flow stresses were obtained as the sum of different contributions as given by Equation (5). The effective and the athermal stresses are reported in Figure 11a,c and Figure 11b,d, respectively. The strain rate has no influence on them under 150 °C since the flow stress has no dependency on the strain rate at low deformation temperatures.

The athermal stress grows rapidly below 200 °C, and more slowly when the temperature increases. Saturation of the stress is observed at low strain rates and moderate to high temperatures. This is because the static part of the evolution rates is not negligible under these deformation conditions. The effective stress saturates at all temperatures and strain rates. The temperature has no influence below 100 °C. Above 100 °C the curves are regularly spaced, following the linearity of Equation (7). The contribution of the intermetallic phases to the resolved shear stress $\tau_{d}$ are assumed to be constant for each temperature since the volume and size of precipitates considered for its calculation (small Mn and Fe rich aluminides) do not change. The values of $\tau_{d}$ decrease linearly with the temperature from 8.2 MPa at 25 °C to 6.5 MPa at 400 °C.

## 4. Discussion and Conclusions

**Figure 12:**
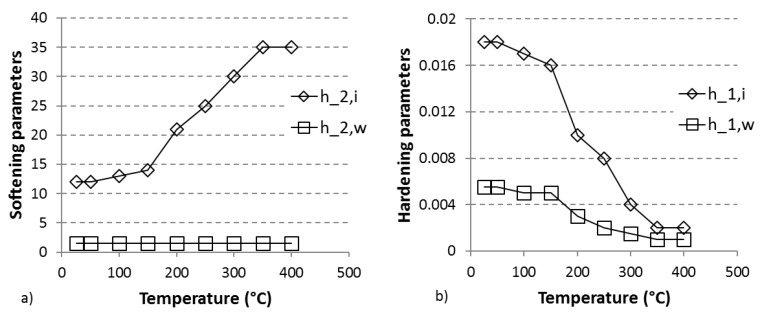
Softening (**a**) and hardening (**b**) parameters as a function of the temperature for dislocation densities walls (w) and interiors (i).

The flow stress model uses a relatively common approach to constitutive modelling. A strong hypothesis, based on the Orowan equation, is that the mobile dislocation density depends only on the strain rate, and remains constant during deformation at constant strain rate. The dislocation rate parameters are plotted against the temperature in Figure 12. The decrease of the hardening parameter $h_{1,i}$ with the temperature indicates that the storage of dislocations becomes less effective when the temperature increases, that is, that the mobile dislocations travel a longer distance before being immobilised, indicating an improved capacity for bypassing local obstacles when the temperature increases. The results also indicate that the hardening due to subgrain formation is promoted at lower temperatures since $h_{1,w}$ decreases with increasing temperatures. As expected, $h_{2,i}$ increases with the temperature, meaning that the dynamic recovery of cell interior dislocations is promoted. Interestingly, $h_{2,w}$ does not depend on the temperature. This seems to indicate that the wall dislocations, being already arranged in a configuration of low energy, are not appreciably affected by dynamic climb.

The evolution of grain size after recrystallisation in samples deformed by plane strain compression can be explained as follows. As the critical bulge size for Strain Induced Boundary Migration has an inverse relationship to the stored energy of deformation, more grains can nucleate in the middle of the deformation crosses, where the accumulated strain is larger, than in regions of lower strain. The new grains impinge on each other upon growth and limit the grain size in the deformation crosses. Furthermore, the recrystallised grains are elongated in the direction of the alignment of the large aluminides. This is especially obvious during annealing at 300 °C between the deformation crosses. The grain growth in the transverse direction is severely limited in comparison to the rolling direction. In those regions where the intermetallic phases are located, the pinning effect on the moving boundaries increases accordingly, enough to stop them. At higher deformations and annealing temperatures, the same trend would be observed if the grains did not impinge on each other before reaching the bigger aluminides. The small aluminides, homogeneously distributed, might still slow down the boundary migration but cannot completely pin the grain boundaries during recrystallisation. Within the time frame of the measurements, the grain size appears to be stabilised. As grain growth after recrystallisation is driven only by capillarity effects, the pressure on the boundaries is much lower than during recrystallisation. Pinning by the homogeneously distributed smaller aluminides can then effectively counter balance this pressure and prevent any further boundary movement. Although above 350 °C, the Mg_2_Si phase dissolves, it does not affect the recrystallisation kinetics, because these particles are too coarse and too widely spaced to effectively pin the boundaries. The effects of the finely dispersed aluminides and of the lines of coarse aluminides are dominant. The most critical parameter in determining the final grain size appeared to be density of nuclei. The more grains nucleate, the less space they must grow and the lower the final grain size. By slightly increasing the density of nuclei being created, the predicted grain radius can be decreased to match the experimental measurements.

## Figures and Tables

**Table 1:** Chemical composition of the commercial AA6082, in weight percent.

Si	Fe	Cu	Mn	Mg	Cr	Ni	Zn	Ti	Al
0.88	0.39	0.07	0.43	0.81	0.02	0.01	0.04	0.04	Balance.
